# Molecular and behavioral profiling of *Dbx1*-derived neurons in the arcuate, lateral and ventromedial hypothalamic nuclei

**DOI:** 10.1186/s13064-016-0067-9

**Published:** 2016-05-21

**Authors:** Katie Sokolowski, Tuyen Tran, Shigeyuki Esumi, Yasmin Kamal, Livio Oboti, Julieta Lischinsky, Meredith Goodrich, Andrew Lam, Margaret Carter, Yasushi Nakagawa, Joshua G. Corbin

**Affiliations:** Center for Neuroscience Research, Children’s National Medical Center, 111 Michigan Avenue, NW, Washington, 20010 DC USA; Department of Morphological Neural Science, Graduate School of Medical Sciences, Kumamoto University, Kumamoto, 860-8556 Japan; Institute for Biomedical Sciences, The George Washington University, Washington, 20037 DC USA; Department of Neuroscience, University of Minnesota Medical School, Minneapolis, 55455 MN USA

**Keywords:** *Dbx1*, Hypothalamus, Behavior, Neuronal fate, Neuronal development

## Abstract

**Background:**

Neurons in the hypothalamus function to regulate the state of the animal during both learned and innate behaviors, and alterations in hypothalamic development may contribute to pathological conditions such as anxiety, depression or obesity. Despite many studies of hypothalamic development and function, the link between embryonic development and innate behaviors remains unexplored. Here, focusing on the embryonically expressed homeodomain-containing gene *Developing Brain Homeobox 1* (*Dbx1*), we explored the relationship between embryonic lineage, post-natal neuronal identity and lineage-specific responses to innate cues. We found that *Dbx1* is widely expressed across multiple developing hypothalamic subdomains. Using standard and inducible fate-mapping to trace the *Dbx1*-derived neurons, we identified their contribution to specific neuronal subtypes across hypothalamic nuclei and further mapped their activation patterns in response to a series of well-defined innate behaviors.

**Results:**

*Dbx1*-derived neurons occupy multiple postnatal hypothalamic nuclei including the lateral hypothalamus (LH), arcuate nucleus (Arc) and the ventral medial hypothalamus (VMH). Within these nuclei, *Dbx1*^+^ progenitors generate a large proportion of the Pmch-, Nesfatin-, Cart-, Hcrt-, Agrp- and ERα-expressing neuronal populations, and to a lesser extent the Pomc-, TH- and Aromatase-expressing populations. Inducible fate-mapping reveals distinct temporal windows for development of the *Dbx1*-derived LH and Arc populations, with Agrp^+^ and Cart^+^ populations in the Arc arising early (E7.5-E9.5), while Pmch^+^ and Hcrt^+^ populations in the LH derived from progenitors expressing *Dbx1* later (E9.5-E11.5). Moreover, as revealed by c-Fos labeling, *Dbx1*-derived cells in male and female LH, Arc and VMH are responsive during mating and aggression. In contrast, *Dbx1*-lineage cells in the Arc and LH have a broader behavioral tuning, which includes responding to fasting and predator odor cues.

**Conclusion:**

We define a novel fate map of the hypothalamus with respect to *Dbx1* expression in hypothalamic progenitor zones. We demonstrate that in a temporally regulated manner, *Dbx1*-derived neurons contribute to molecularly distinct neuronal populations in the LH, Arc and VMH that have been implicated in a variety of hypothalamic-driven behaviors. Consistent with this, *Dbx1*-derived neurons in the LH, Arc and VMH are activated during stress and other innate behavioral responses, implicating their involvement in these diverse behaviors.

**Electronic supplementary material:**

The online version of this article (doi:10.1186/s13064-016-0067-9) contains supplementary material, which is available to authorized users.

## Background

The hypothalamus plays a critical role in a variety of behaviors essential for survival, species propagation and maintenance of homeostasis. These behaviors most prominently include regulation of hunger and body temperature states, mating, aggression and appropriate responses to threatening encounters [1–5]. Proper development of this system is critical for normal function and behavior, and altered trajectories of development may potentially contribute to diseases or disorders such as obesity, depression and anxiety [4, 6].

At the anatomical level, the hypothalamus is composed of multiple nuclei each characterized largely by their location in the rostral-caudal plane, molecularly and functionally distinct diversity of neuronal cell types and involvement in different aspects of behaviors. Different nuclei, as well as the constellation of neuronal subtypes, have specific and overlapping roles in coordinating complex animal behaviors. For example, the arcuate nucleus (Arc) is linked mostly to regulating hunger and satiety states [2, 7]. The primary role of the lateral hypothalamus (LH) is to create a state of arousal [8, 9], and as such is implicated in diverse behaviors such feeding/food seeking, mating, aggression and predator threat responses. Another major nucleus is the ventromedial hypothalamus (VMH), which appears to be involved in, if not critical for, aggressive and reproductive behaviors as well as innate fear responses [1, 10, 11].

These diverse behaviors appear to be regulated by different molecularly identifiable neuronal subpopulations located within these distinct nuclei [12–15]. Neuronal identity of mature hypothalamic neurons is presumably endowed during embryonic development through the combinatorial expression of both distinct and overlapping sets of transcription factors [16–18]. While the complete developmental blueprint for specification of different hypothalamic neuronal subpopulations remains to be elucidated, some of the key intrinsic and extrinsic factors controlling specification and differentiation have begun to be elucidated [18]. In our recent studies, we revealed that the homeodomain-containing transcription factor encoding gene *Dbx1* acts as a putative selector gene for the generation of the pro-melanin-concentrating hormone (Pmch)^+^, Calbindin^+^, and orexin/hypocretin (Hcrt)^+^ neurons in the LH and Agouti-related peptide (Agrp)/ Neuropeptide Y (Npy)^+^ neurons in the Arc [19]. At the behavioral level, we further found that *Dbx1* is required for hypothalamic-mediated feeding and stress responses in adult animals. However, despite this understanding of *Dbx1* gene function, the contribution of *Dbx1*-derived neurons to different hypothalamic neuronal populations and activation of *Dbx1*-derived neurons during innate behaviors currently remains unknown.

To determine the contribution of the *Dbx1*-lineage to distinct neuronal subpopulations within LH, Arc and VMH, by using both standard and inducible fate-mapping techniques we significantly expand upon existing *Dbx1* lineage studies conducted by the Allen Institute for Brain Science (Allen Brain Atlas experiment 167643944). We found that within these regions, *Dbx1*-derived neurons contribute to diverse populations of molecularly identified neuronal subpopulations. Moreover, we characterized the behavioral activation patterns of *Dbx1*-derived neurons in the LH, Arc and VMH after exposure to a broad array of innate behavioral cues. We found that *Dbx1*-derived neurons are activated by predator odor, fasting, mating and male aggression, without specific tuning toward one behavior. Therefore, across hypothalamic nuclei *Dbx1*-derived neurons likely play a critical role in regulation of different innate behaviors.

## Methods

### Animals

Mice were housed in the temperature- and light-controlled Children’s National Medical Center animal care facility and given food and water *ad libitum*, unless otherwise stated. All animal procedures were approved by Children’s National Medical Center’s Institutional Animal Care and Utilization Committee (IACUC) and conformed to NIH Guidelines for animal use.

### Fate mapping

*ROSA*^*loxP-STOP-loxP-YFP*^ (*RYFP*) reporter mice were obtained from Jackson Labs (stock no: 006148). *Dbx1*^*Cre*^ mice were kindly provided by Dr. Alessandra Pierani [20]. *Dbx1*^*Cre*^*ERT2* mice were previously generated in the Corbin lab [21] (available at Jackson Labs, stock no: 028131). For fate-mapping analysis *Dbx1*^*Cre+/-*^;*RYFP*^*+/-*^ mice were obtained by crossing *Dbx1*^*Cre+/-*^ males with *RYFP*^*+/+*^ females. For genetically inducible fate-mapping analysis *Dbx1*^*CreERT2+/-*;^*RYFP*^*+/-*^ mice were obtained by crossing *Dbx1*^*CreERT2+/-*;^males with *RYFP*^*+/+*^ females. Mice were genotyped by Transnetyx Inc. Genotyping Services (Cordova, TN). Noon on the day of vaginal plug was designated as E0.5. For inducible fate mapping, tamoxifen (Sigma T5648) was dissolved in sesame oil (Sigma S3547) to a concentration of 3 mg/ml. Pregnant females were given 100 μl (0.3 mg of tamoxifen; approximately 0.1 mg/grams body weight) via oral gavage with animal feeding syringes (FisherBrand 01-208-87).

### Tissue processing, *in situ* hybridization (ISH) and immunohistochemisty (IHC)

For ISH, embryos were dissected and fixed in 4 % paraformaldehyde overnight and then cryoprotected in 30 % sucrose. Embryonic brains were sectioned on a cryostat at 20 μm. Every tenth section was collected in a set, for a total of ten sets of sections representing the entire brain. One probe was run on one set of sections; 10 probes were run on serial sections from the same brains. RNA *in situ* hybridization was performed as previously described [19]. cDNA plasmids were obtained from Drs. Seth Blackshaw (*Pmch* and *Hcrt*), Paul Gray (*Agrp* and *Pomc*), Yasushi Nakagawa (*Lhx9*), Chen-Ming Fan (*Sim1*), Kenneth Campbell (*Dbx1*), and Kazue Hashimoto-Torii (*Nr5a1* and *Fezf1*). Images were taken on an Olympus BX51 at 4x and 10x.

For IHC, postnatal day (P) 21 mice were perfused with 4 % paraformaldehyde. Postnatal brains were sectioned at 50 μm on a vibratome (Leica VT1000 S). Every sixth section was collected in one well, with six wells containing every section from the brain. Embryonic brains were processed as described above and used for IHC. Immunofluorescent staining on sections was performed as previously described [19]. Primary antibodies used were: rat anti-GFP (1:1000, 04404-84, Nacalai, Kyoto Japan), goat anti-Pomc (1:100, ab322893, Abcam), goat anti-Pmch (1:1000, sc-14509, Santa Cruz), goat anti-Agrp (1:1000, AF634, R&D Systems), rabbit anti-c-Fos (1:1000, sc52, Santa Cruz), rabbit anti-Hcrt (1:1000, AB3096, Millipore), rabbit anti-TH (1:500, sc14007, Santa Cruz), rabbit anti-Cart (1:20,000, 55-102, Phoenix Pharmaceuticals), sheep anti-Nesfatin (1:500, AF6895, R&D Systems) and rabbit anti-Dbx1 (1:100) [22]. Secondary antibodies were: donkey anti-rat IgG Alexa 488 (1:1000 Invitrogen), donkey anti-rabbit Cy3 or Cy5 (1:1000 Jackson ImmunoResearch), donkey anti-goat Cy3 or Cy5 (1:1000 Jackson ImmunoResearch) and donkey anti-sheep Cy3 or Cy5 (1:1000 Jackson ImmunoResearch). Co-labeling was determined after images were taken on an Olympus FV1000 confocal microscope at 20x or 40x magnification on an optical slice of 1–3 μm.

Analysis of double labeled IHC stained sections was performed on every sixth section of the brain containing the region of interest. LH, Arc and VMH were defined from Bregma -1.06 to -1.94, -1.22 to -2.06 and -1.34 to -1.70, respectively, using the Paxinos and Watson anatomical atlas. DAPI (DAPI Fluoromount-G, SouthernBiotech #0100-20) was used as a nuclear counterstain to aid the definition of hypothalamic nuclei. Quantification of c-Fos and co-labeled cells in LH, Arc and VMH were analyzed across 3, 2 and 1 sections, encompassing each nuclei, per animal, respectively. The average total number of YFP^+^ cells counted per animal was 1027, 546, and 1055 in the LH, Arc, and VMH, respectively. Cell counts were expressed as the number of neurons/mm^2^ for each animal. Animals within a group were then averaged.

### Behavioral paradigms

Unless otherwise stated, all animals were group-housed by sex after weaning and then singly housed and habituated to the behavioral assay 1 week prior to experiment, which took place >1 h after the beginning of the dark cycle. Mating, aggression, predator avoidance and fasting assays were performed in adult mice (P40-50) as previously reported [19]. Animals were sacrificed 1 h after the start of the behavioral paradigm and brains processed for c-Fos and YFP IHC.

### Statistical evaluation

Quantitation of data was performed blind to relevant variables. Using GraphPad Prism 6 statistical software, a One-way ANOVA followed by Tukey–Kramer multiple comparison test was used for analysis of experiments involving three groups with one comparison (Figs. 6 and 7, comparing cell counts at 3 different labeling periods: TME7.5, 9.5, and 11.5), and an unpaired *t*-test with Welch’s correction was used for analysis of experiments involving two groups.

## Results

### Regional fate of *Dbx1*-derived neurons

**Fig. 1:**
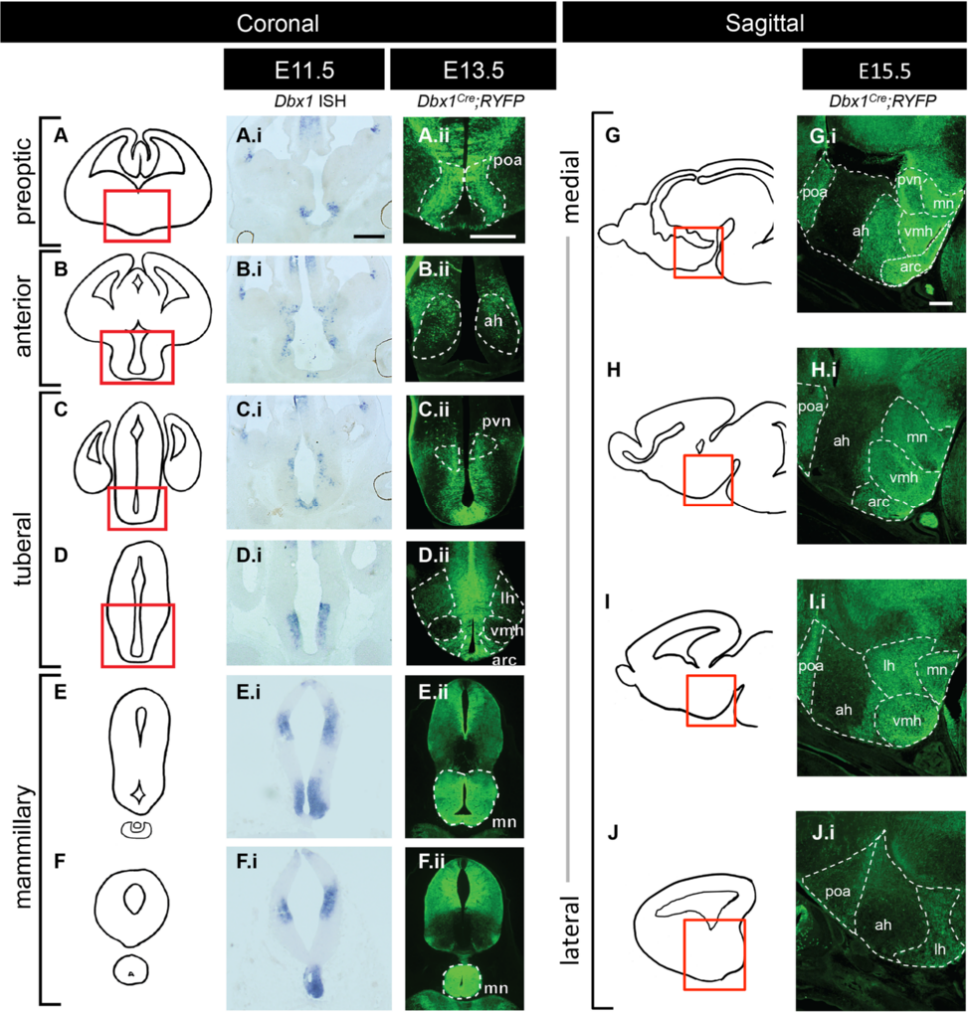
Hypothalamic *Dbx1* expression and fate. **a**-**f** Schematic of rostral (*top*) to caudal (*bottom*) coronal views of the embryonic forebrain. **g**-**j** Schematic of medial (*top*) to lateral (*bottom*) sagittal views of the embryonic forebrain. (A.i-F.i) As shown by ISH at E11.5, *Dbx1* is expressed throughout the rostral-caudal extent of the developing hypothalamus including in the preoptic, anterior, tuberal and mammillary progenitor domains. (A.ii-F.ii; G.i-J.i) As shown by YFP expression in E13.5 (A.ii-F.ii) and E15.5 (G.i-J.i). *Dbx1* ^*Cre*^ *;Rosa26YFP* embryos, *Dbx1*-derived cells emerge from progenitor domains and coalesce in multiple emerging hypothalamic nuclei. Abbreviations: ah (embryonic anterior hypothalamus); arc (embryonic arcuate nucleus); lh (embryonic lateral hypothalamus); mn (embryonic mammillary nucleus); poa (embryonic postnatal preoptic area); pvn (embryonic paraventricular nucleus); vmh (embryonic ventral medial hypothalamus). The scale bar represents 500 *μ*m for coronal (**a**-**f**) and sagittal (**g**-**j**) sections

Previous studies by us and others revealed that *Dbx1* is expressed in the ventricular zone (VZ) of the ventral diencephalon, the primordium of the hypothalamus, during early to mid-neurogenesis, with downregulation of expression as precursors transit out of the VZ [19, 23–26]. Using criteria previously established for emerging hypothalamic domains [27, 28], we observed that *Dbx1* was expressed across the preoptic, anterior, tuberal and mammillary regions (Fig. 1A.i-F.i). Because *Dbx1* expression is downregulated as cells as cells become post-mitotic, we examined the hypothalamic fate of *Dbx1*-derived neurons by crossing previously generated *Dbx1*^*Cre*^ knockin mice [20] to *Rosa26*^*lox-STOP-lox-YFP*^ reporter mice. This mouse faithfully recapitulates the pattern of *Dbx1* expression across the neuraxis and thus provides a validated tool to study the *Dbx1*-lineage (Additional file 1: Figure S1 and [19–21]). Here, we show that *Dbx1*-derived neurons contributed to multiple developing hypothalamic nuclei including the primordial LH, Arc, VMH, preoptic area, anterior hypothalamus, paraventricular nucleus and mammillary nuclei (Fig. 1A.ii-F.ii). To correlate location of recombined cells with emerging hypothalamic nuclei, we carried out *in situ* hybridization for specific markers (*Pomc*, *Bsx*, *Fezf1*, *Nr5a1*, *Pmch*, *Lhx9*, and *Sim1*; Additional file 2: Figure S2) that define either specific cell populations or individual nuclei. These analyses revealed that *Dbx1*-derived cells contributed to multiple emerging hypothalamic nuclei/subdomains.

**Fig. 2:**
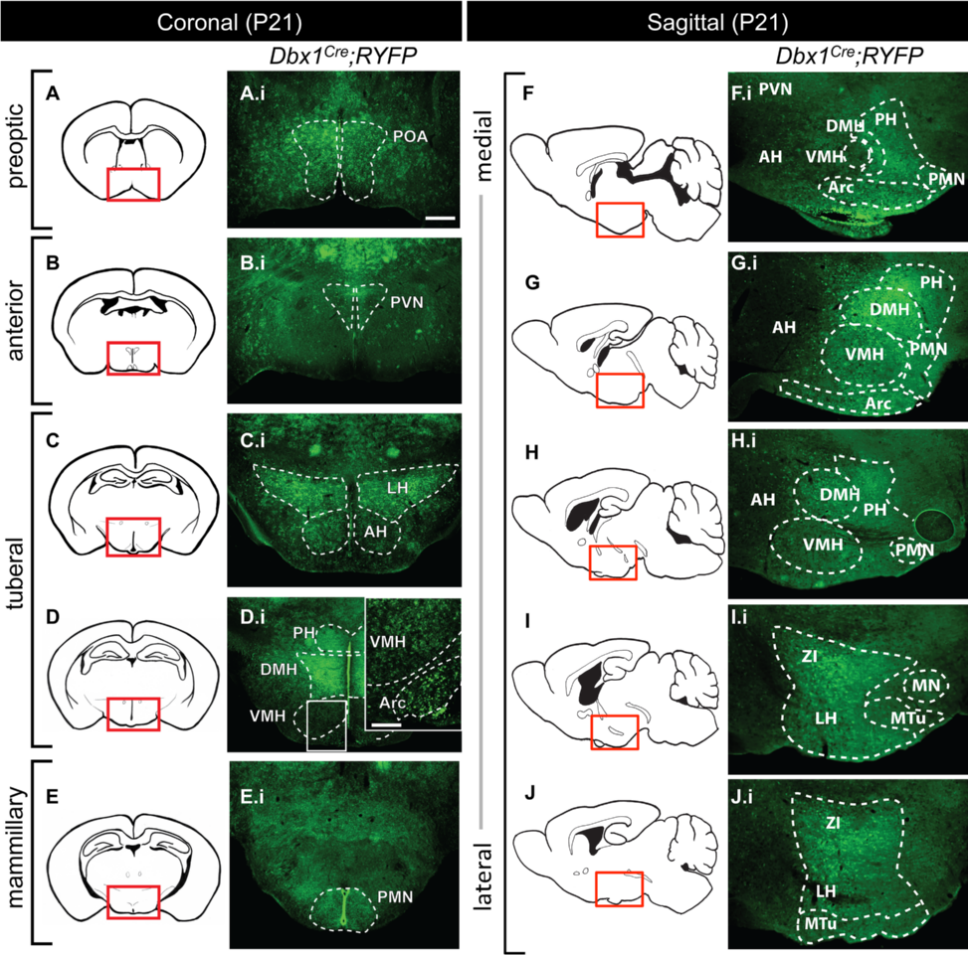
Hypothalamic *Dbx1* expression and fate. **a**-**j** Schematic of rostral (*top*) to caudal (*bottom*) coronal (**a**-**e**) or medial (*top*) to lateral (*bottom*) sagittal (**f**-**j**) views of the postnatal forebrain. (A.i-J.i) In the postnatal forebrain (P21) *Dbx1*-derived cells are found in numerous hypothalamic nuclei. Abbreviations: AH (anterior hypothalamus); Arc (arcuate nucleus); DMH (dorsomedial hypothalamus); LH (lateral hypothalamus); MN (mammillary nucleus); MTu (medial tuberal nucleus); PH (postnatal posterior hypothalamus); POA (preoptic area); PMN (premammillary nucleus); PVN (paraventricular nucleus); VMH (ventral medial hypothalamus); ZI (zona incerta). The scale bar represents 500 *μ*m

To investigate the spatial residence of *Dbx1*-derived cells in the postnatal hypothalamus, we analyzed coronal and sagittal tissue sections from P21 fate-mapped mice. *Dbx1*-derived cells in the hypothalamus appeared to be primarily neurons, with an estimated <10 % differentiating into glia based on morphological criteria (data not shown). Consistent with the embryonic expression pattern, *Dbx1*-derived cells occupied all domains of the P21 hypothalamus including the preoptic, anterior, tuberal, and mammillary domains, with the greatest apparent number of fate-mapped cells residing in the preoptic, tuberal and mammillary domains (Fig. 2), patterns which were similarly observed in the P40-50 hypothalamus (data not shown and Fig. 8). Large numbers of *Dbx1*-derived cells were specifically observed in the following anterior to posterior located nuclei: Arc, LH, preoptic area, paraventricular nucleus, zona incerta (which may be derived from the pre-thalamus [22]), dorsomedial hypothalamus, medial tuberal nucleus, posterior hypothalamus, pre-mammillary and mammillary nuclei. *Dbx1*-derived cells were also present in large numbers in the VMH, although with relatively lower intensity of YFP expression. The anterior hypothalamus had very few *Dbx1*-derived cells.

### Identity of *Dbx1*-derived neurons

**Fig. 3:**
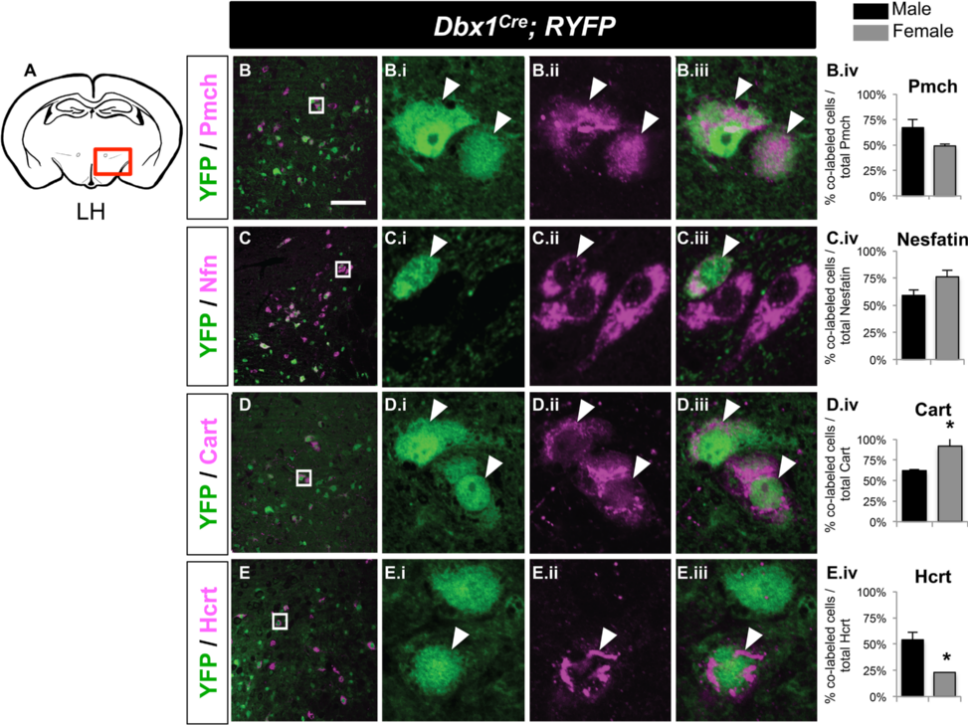
Postnatal fate of *Dbx1*-derived cells in the LH in males and females. Schematic of a coronal view of the postnatal brain at the level of the LH (**a**) show regions analyzed in (B-E.iv). Low magnification merged panels (**b**, **c**, **d**, and **e**) are shown on the left, with high magnification of boxed area shown in right panels (i - iii). In the LH, a high percentage of *Dbx1*-derived neurons (*green*) express markers (*red*) Pmch (B-B.iv), Nesfatin (Nfn; C-C.iv), Cart (D-D.iv) or Hcrt (E-E.iv). The *Dbx1*-lineage contribution to the Cart (D.iv) and Hcrt (E.iv) populations is sexually dimorphic. Mean ± SEM; *n* = 3–6 mice per sex; *, *p* < 0.05. The scale bar represents 50 *μ*m for low mag panels (**b**, **c**, **d**, and **e**) and 10 *μ*m for high mag panels (i-iii)

**Fig. 4:**
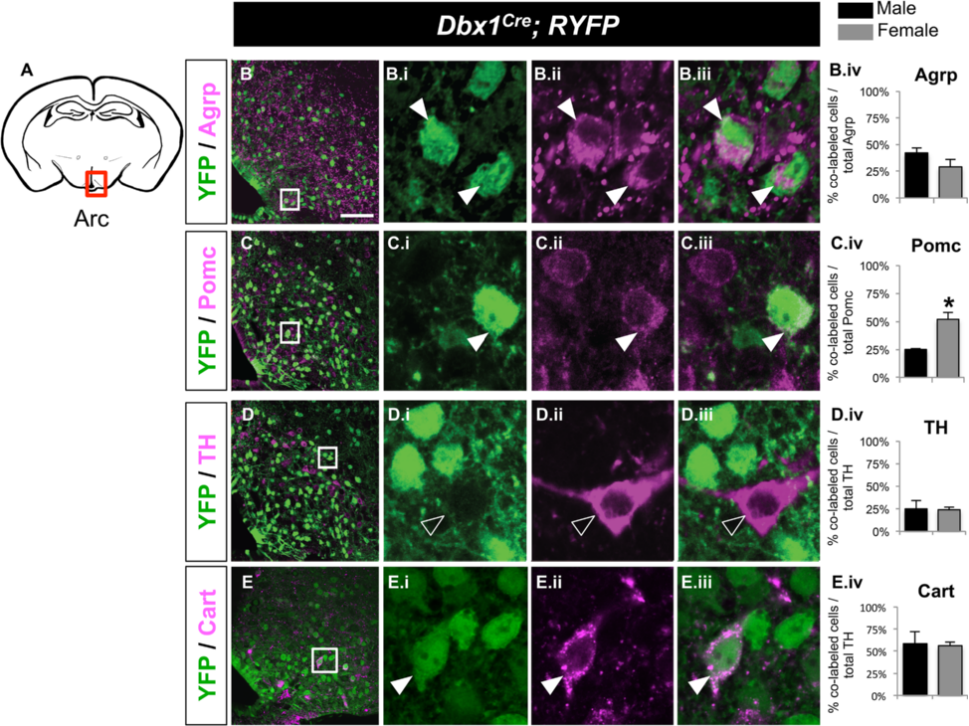
Postnatal fate of *Dbx1*-derived cells in the LH and Arc in males and females. Schematic of a coronal view of the postnatal brain at the level of the Arc (**a**) show regions analyzed in (B-E.iv). Low magnification merged panels (**b**-**e**) are shown on the left, with high magnification of boxed area shown in right panels (i - iii). In the Arc, subpopulations of *Dbx1*-derived neurons (*green*) express markers (*red*) Agrp (B-B.iv), Pomc (C-C.iv), TH (D-D.iv), or Cart (E-E.iv). The *Dbx1*-lineage contribution to the Pomc (C.iv) populations is sexually dimorphic. Mean ± SEM; *n* = 3–6 mice per sex; *, *p* < 0.05. The scale bar represents 50 *μ*m for low mag panels (**b**-**e**) and 10 *μ*m for high mag panels (i-iii)

**Fig. 5:**
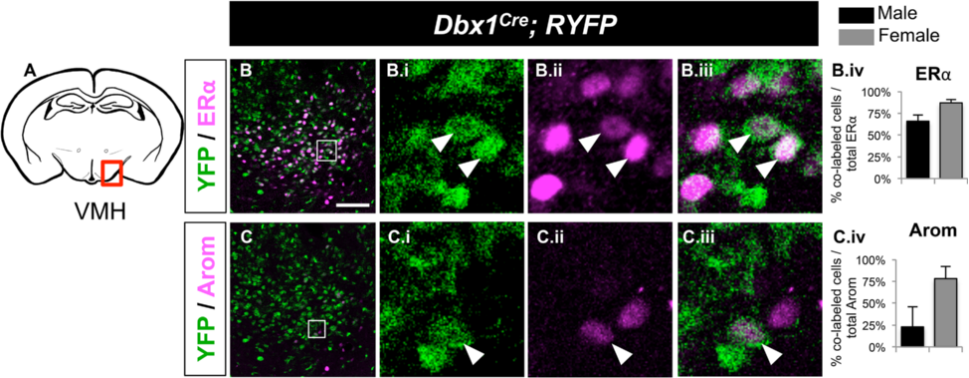
Postnatal fate of *Dbx1*-derived cells in the VMH in males and females. Schematic of a coronal view of the postnatal brain at the level of the VMH (**a**) show regions analyzed in (B-C.iv). Low magnification merged panels (**b** and **c**) are shown on the left, with high magnification of boxed area shown in right panels (i - iii). In the VMH, a high percentage of *Dbx1*-derived neurons (*green*) express markers (*red*) Estrogen Receptor (ER; B-B.iv) and Aromatase (Arom; C-C.iv). Mean ± SEM; *n* = 3 mice per sex. The scale bar represents 50 *μ*m for low mag panels (**b** and **c**) and 10 *μ*m for high mag panels (i-iii)

Our previous studies revealed that *Dbx1* acts as a putative selector gene to specify the Hcrt^+^ and Pmch^+^ neurons of the LH and Agrp^+^/Npy^+^ neurons of the Arc, without altering select markers in the VMH [19]. Hcrt^+^ and Pmch^+^ neurons control arousal states associated with sleep/wake, stress and feeding [29–31]. In the Arc, feeding is controlled in part by the opposing actions of Agrp- and Pro-opiomelanocortin- (Pomc) expressing neurons [2, 32, 33]. To examine the specific contributions of *Dbx1*-derived hypothalamic neurons to these critical LH, Arc and VMH populations, we conducted co-expression analyses of YFP and markers of LH and Arc neuronal subtypes (Figs. 3, 4, and 5). Details of the findings are described below.

#### Lateral hypothalamus

Pmch-expressing neurons are one of the two major output populations of the LH and respond to high concentrations of blood glucose [30]. Pmch^+^ neurons are defined by their co-expression with Nesfatin (Nfn)- and Cocaine and amphetamine regulated transcript (Cart) [34–36]. Our previous *Dbx1* loss-of-function studies revealed that Pmch neurons are specified by *Dbx1*-dependent mechanisms [19]. Here we show that the majority of Pmch-, Nfn- and Cart- expressing neurons were *Dbx1*-derived (Fig. 3B-D.iv). This finding combined with our previous loss-of-function studies is consistent with the hypothesis that *Dbx1* acts cell autonomously to specify the fate of Pmch-, Nfn- and Cart- expressing neurons in the LH. Interestingly, the contribution of *Dbx1*-derived neurons to the Cart^+^ and Hcrt^+^ populations differed between males and females, revealing sexually dimorphic contributions of the *Dbx1*-lineage within the LH.

The other major neuronal output population of the LH is defined by expression of Hcrt, which, in contrast to the Pmch^+^ population, is activated in the presence of low glucose [30]. Hcrt^+^ neuron specification, differentiation and number appear to require *Lhx9* and histamine expression [37, 38]. Here, we reveal that the Hcrt-expressing population was at least partially *Dbx1*-derived, and also differed between males and females (Fig. 3E-E.iv).

#### Arcuate nucleus

In the Arc, three major neuronal populations are distinguished by their expression of Agrp, Pomc/Cart or tyrosine hydroxylase (TH) [39–41]. The expression of *Bsx* drives immature neurons towards a mature appetite-stimulating (orexigenic) Agrp^+^ fate, but not other Arc cell types such as the appetite-inhibiting (anorexigenic) Pomc neurons [42]. Our previous studies in *Dbx1* loss-of-function mice demonstrated an approximately 50 % reduction in *Bsx* and *Agrp* expression, with no changes in *Pomc* or *TH* expression [19]. Our analysis here revealed that *Dbx1*-derived neurons contributed to all three populations, although at varying levels (Fig. 4). *Dbx1*-derived neurons contributed to 29–42 % of the Agrp^+^ population in males and females (Fig. 4B-B.iv). The Pomc-, TH- and Cart-expressing populations were 24–44 % *Dbx1*-derived, with the exception of the Pomc population in females, which was 52 % ± 6 % *Dbx1*-derived (mean ± SEM; Fig. 4C-E.iv). Of these Arc populations, the contribution of the *Dbx1*-derived lineage to the Pomc population was sexually dimorphic, with a greater contribution to Pomc^+^ neurons in females compared to males. Thus, the three major populations in the Arc are also derived from *Dbx1*-expressing progenitors.

#### Ventromedial hypothalamus

Subsets of neuronal populations in the VMH can be defined by their expression of the sex steroid pathway markers Estrogen Receptor alpha (ERα) and Aromatase (Arom), which are known to function in mating and aggressive behaviors in mice [14, 43–45]. The majority of ERα neurons were *Dbx1*-derived (mean ± SEM; male: 66 % ± 7 %, female: 87 % ± 4 %; Fig. 5B-B.iv). In contrast, more variable results were observed with the Arom^+^ population, which depended on the sex of the animal. While the majority of the Arom^+^ population in females was *Dbx1*-derived, there was an apparently less, albeit more variable contribution of the *Dbx1*-lineage in males (male: 23 % ± 23 %, female: 78 % ± 14 %; Fig. 5C-C.iv). Overall, although varying by sex, these results show significant contribution of the *Dbx1*-lineage to the VMH ERα- and Arom-expressing populations.

### Temporal labeling of *Dbx1*-derived neurons

**Fig. 6:**
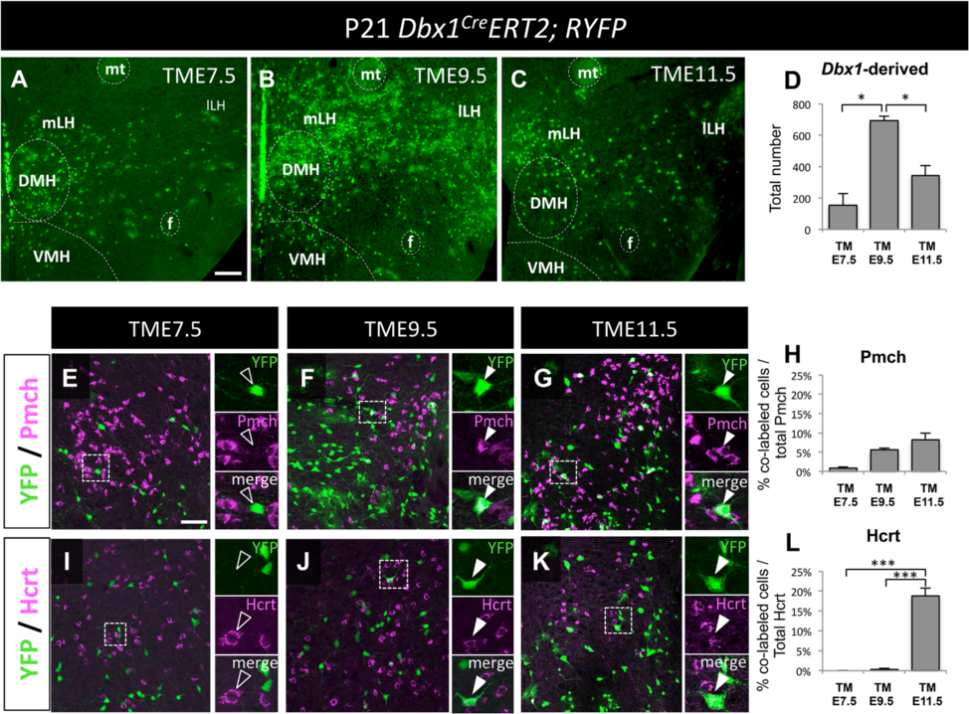
Temporal-dependent fate of *Dbx1*-derived cells in the LH. **a**-**d** Images of the hypothalamus with YFP-expressing cells in medial and lateral portions of the LH after tamoxifen gavage at E7.5 (**a**), E9.5 (**b**), and E11.5 (**c**). **e**-**l** Proportionately more Pmch (**e**-**h**) and Hcrt (**i**-**l**) co-labeled with YFP when tamoxifen was given during later stages of development (E9.5-E11.5). Mean ± SEM; E7.5 *n* = 4 mice (2 males, 2 females); E9.5 *n* = 3 mice (0 males, 3 females); and E11.5 *n* = 9 mice (2 males, 7 females). The scale bar represents 250 *μ*m for panels (**a**-**c**), 50 *μ*m for low mag and 10 *μ*m images in panels (**e**-**k**). Abbreviations: mt, mammillothalamic tract; f, fimbria; VMH, ventromedial hypothalamic nucleus; mLH, medial portion of the lateral hypothalamus; lLH, lateral portion of the lateral hypothalamus; DMH, dorsomedial hypothalamic nucleus

To investigate the correlation between timing of *Dbx1* expression in relation to the spatial localization of *Dbx1*-derived neurons across the hypothalamus, we delivered tamoxifen to pregnant *Dbx1*^*CreERT2*^*;Rosa26*^*lox-STOP-lox-YFP*^ mice at early (TME7.5), middle (TME9.5) and later (TME11.5) stages of gestation. CreER has been shown to be translocated into the nucleus within 6 h of tamoxifen administration and is sustained for 24 to 36 h [46, 47]. We observed that fate-mapped cells derived from the progenitors expressing *Dbx1* at early stages (TME7.5) populate medial Arc and periventricular regions, while progenitors expressing *Dbx1* at mid stages (TME9.5) occupy dorsal-lateral regions, with the ventral-lateral and paraventricular regions settled by neurons marked at later stages (TME11.5) (Fig. 6a-d). This timing of development is reminiscent of findings from a previous temporal fate mapping study of *Shh*-expressing progenitors [48], and is likely reflective of the dynamic changes in *Dbx1* expression over this developmental window.

Having established that different temporal waves of *Dbx1*-expressing progenitors occupy different post-natal medial to lateral hypothalamic domains, we next focused on development of subpopulations within the LH and Arc, two major nuclei with *Dbx1*-lineage contributions. Previous BrdU labeling studies in rats revealed that Pmch^+^ neurons are born in three major waves, with the early born neurons taking residence in the most lateral regions adjacent to the cerebral peduncle. Cells born later occupy progressively more medial portions of the tuberal mantle. The majority of Pmch^+^ neurons are born during mid stages thus occupying the majority of the zona incerta and LH regions surrounding the fornix [49]. To investigate whether *Dbx1-*derived neurons develop in a similar pattern, we again used inducible fate-mapping and performed double immunohistochemistry. We observed proportionately more double-labeled neurons (Pmch^+^ and YFP^+^) at mid and later stages (TME9.5 and TME11.5; Fig. 6e-h). Thus, development of the Pmch^+^*Dbx1*-derived population follows the same overall pattern of Pmch^+^ development, with the greatest contribution occurring between ~ E10.5-E13.0. Unlike the Pmch^+^ population, less is known about the timing of development of the Hcrt^+^ population. Here, we observed a marked increase in co-labeling of YFP and Hcrt at TME11.5, with little to no co-labeling at earlier stages (Fig. 6i-l). This suggests that the *Dbx1*-derived Hcrt^+^ population observed in Fig. 3E-E.iv most likely arises from a later wave of *Dbx1*^*+*^ progenitors.

**Fig. 7:**
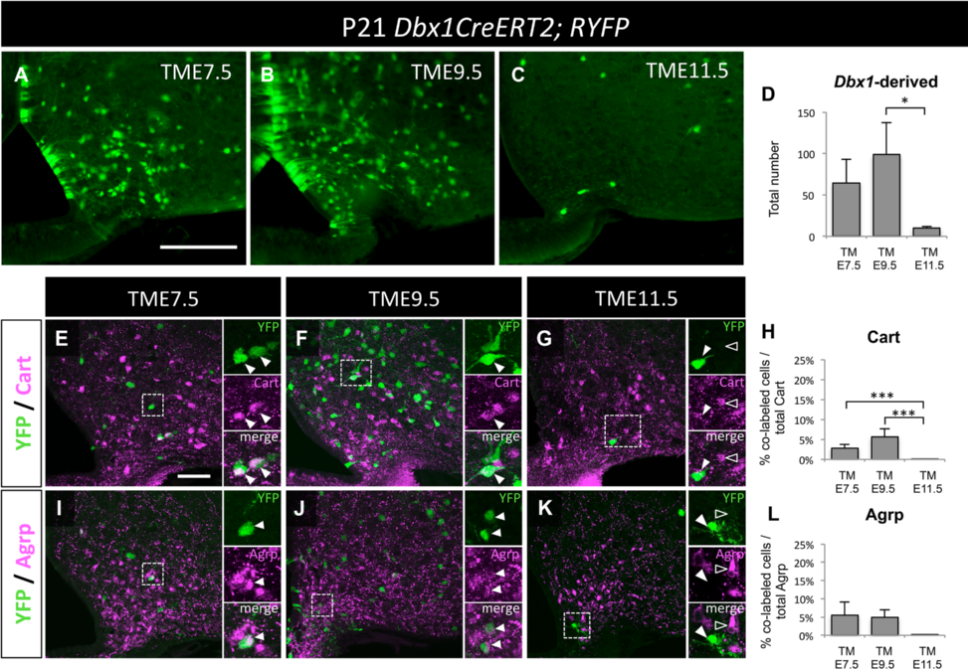
Temporal-dependent fate of *Dbx1*-derived cells in the Arc. **a**-**c** YFP-expressing cells in the Arc after tamoxifen gavage at E7.5 (**a**), E9.5 (**b**), and E11.5 (**c**). Quantification shown in (**d**). **e**-**l** Proportionately more Cart (**e**-**h**) and Agrp (**i**-**l**) co-labeled with YFP when tamoxifen was given during early stages of development (E7.5-E9.5). Mean ± SEM; E7.5 *n* = 4 mice (2 males, 2 females); E9.5 *n* = 3 mice (0 males, 3 females); and E11.5 *n* = 9 mice (2 males, 7 females). The scale bar represents 100 *μ*m for panels (**a**-**c**), 50 *μ*m for low mag and 10 *μ*m images in panels (**e**-**k**)

Compared to the LH, a population of *Dbx1*-derived neurons in the Arc is generated during an earlier wave (Fig. 7a-c). Cart expression defines the Pomc^+^ population in the Arc [40, 41]. The *Dbx1*-lineage contribution to both the non-overlapping Cart- and Agrp-expressing neurons occurs at earlier stages of *Dbx1* expression (Fig. 7e-l). Together these observations demonstrate the temporal contribution of early and late *Dbx1*-expressing progenitors to Arc and LH neuronal populations, with the generation of Arc *Dbx1*-derived populations commencing earlier than LH *Dbx1*-derived populations.

### Activation of *Dbx1*-derived neurons during innate behaviors

Different hypothalamic nuclei are engaged during processing of a variety of innate behaviors such as feeding, mating, aggression and predator odor avoidance [31, 50–54]. Specifically, c-Fos translation occurs in cells of the LH, Arc and VMH in response to fasting, predator odor, conspecific aggression and mating situations [19, 55–58]. Our previous studies revealed that *Dbx1* functions in the specification of the LH and Arc neurons required for physiological and behavioral responses to innate stressors such as stress feeding and predator odor exposure [19]. To investigate the putative involvement of *Dbx1*-derived neurons in different innate behaviors (predator odor, fasting, mating or aggression) we assessed the activation patterns of *Dbx1*-derived neurons in male and female *Dbx1*^*Cre*^*; Rosa26*^*lox-STOP-lox-YFP*^ mice using expression of the immediate early gene c-Fos as a proxy for neuronal activation [59, 60]. We focused our analyses on the LH, Arc and VMH, three major nuclei involved in these select innate behaviors.

#### Lateral hypothalamus

**Fig. 8:**
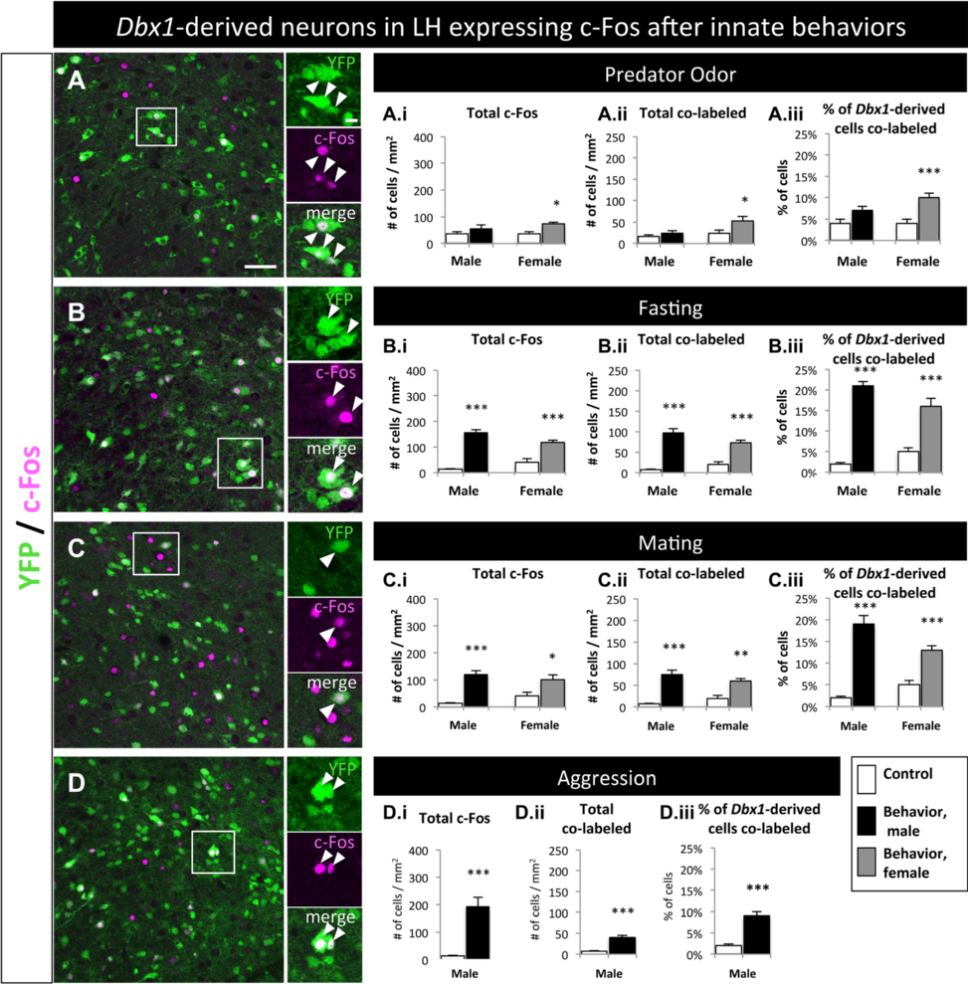
Behavioral involvement of *Dbx1*-derived cells in the LH. **a**-**d** Images of the LH with YFP-expressing cells co-labeled with c-Fos after mice were exposed to predator (**a**), fasting (**b**), mating (**c**), or aggression (**d**) behavioral paradigms. (A.i-D.iii) Quantification of total c-Fos (A.i-D.i), total co-labeled cells (A.ii-D.ii), and percent of *Dbx1*-derived cells co-labeled with c-Fos (YFP + c-Fos / total YFP; A.iii-D.iii) in male and female mice compared to appropriate behavioral controls. Significant increases in total c-Fos, total co-labeled cells, and the percent of *Dbx1*-derived cells expressing c-Fos was detected in both males and females after every behavior, except in males after exposure to rat odor (A.i-A.ii, black bars). Mean ± SEM; *n* = 4–12 mice per subgroup; *, *p* < 0.05; **, *p* < 0.01; ***, *p* < 0.001. The scale bar represents 50 *μ*m for low mag and 10 *μ*m high mag images in panels (**a**-**d**)

The neurons in the LH have various functions during stress, arousal and feeding [29–31]. We placed female and/or male fate-mapped mice in four different behavioral paradigms (predator, fasting, mating and male aggression) and observed a significant increase in c-Fos^+^ cells in the LH in all four behaviors, with the exception of predator odor in males (Fig. 8, Additional file 3: Figure S3A-D.i). We then analyzed the number of c-Fos^+^ neurons that co-labeled with YFP. In all behaviors tested, with the exception of male predator odor, there was a significant increase in the number of co-labeled cells (YFP^+^ and c-Fos^+^; Fig. 8a-d, A.ii-D.ii) as well as the percentage of *Dbx1*-derived cells that co-labeled with c-Fos (YFP^+^ and c-Fos^+^/total YFP^+^; Fig. 8a-d, A.iii-D.iii). This revealed that the *Dbx1*-derived neurons in the LH are active during predator exposure (female), fasting (both sexes), mating (both sexes) and male aggression.

#### Arcuate nucleus

**Fig. 9:**
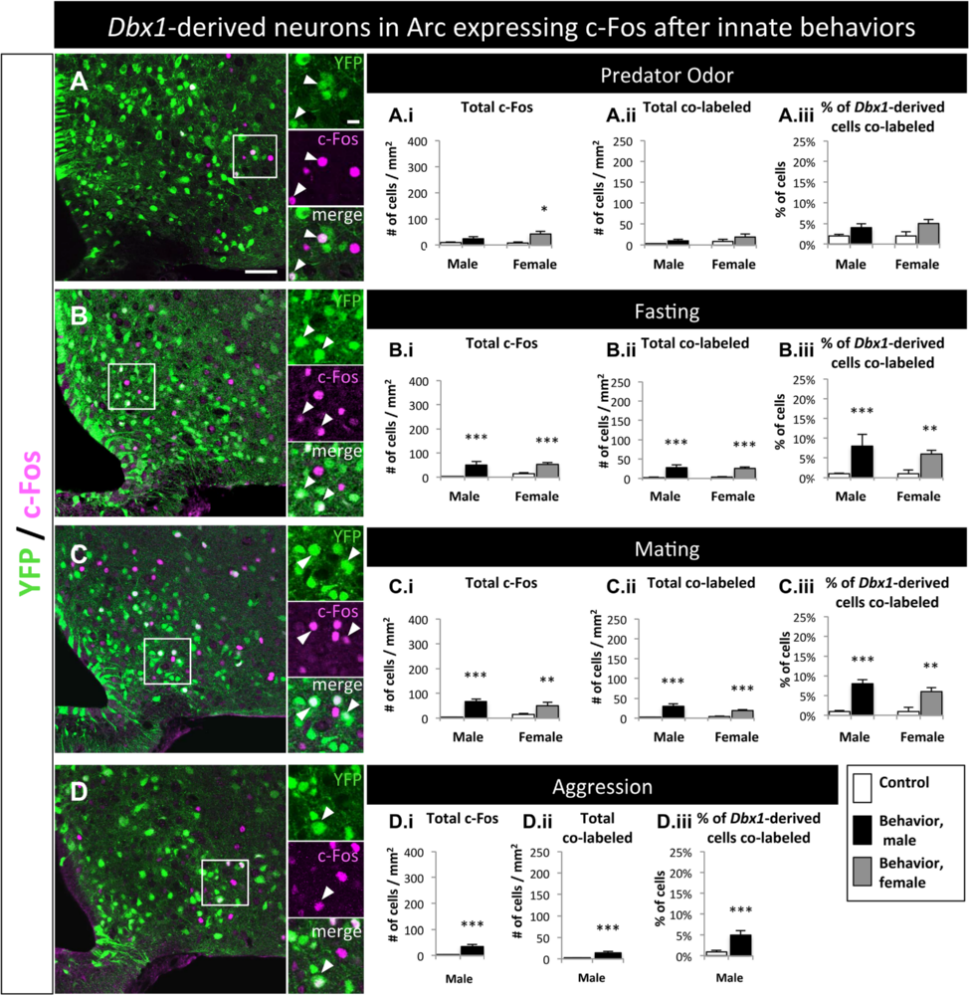
Behavioral involvement of *Dbx1*-derived cells in the Arc. **a**-**d** Images of the Arc with YFP-expressing cells co-labeled with c-Fos after mice were exposed to predator (**a**), fasting (**b**), mating (**c**), or aggression (**d**) behavioral paradigms. (A.i-D.iii) Quantification of total c-Fos (A.i-D.i), total co-labeled cells (A.ii-D.ii), and percent of *Dbx1*-derived cells co-labeled with c-Fos (YFP + c-Fos / total YFP; A.iii-D.iii) in male and female mice compared to appropriate behavioral controls. Significant increases in total c-Fos, total co-labeled cells, and the percent of *Dbx1*-derived cells expressing c-Fos was detected in both males and females after every behavior, except after exposure to rat odor (A.i-A.ii). After exposure to rat odor, only females had a significant increase in total c-Fos in the Arc (A.i, *gray bar*) and only males had a significant increase in the total numbers of co-labeled cells (A.ii, *black bar*). Mean ± SEM; *n* = 4–12 mice per subgroup; *, *p* < 0.05; **, *p* < 0.01; ***, *p* < 0.001. The scale bar represents 50 *μ*m for low mag and 10 *μ*m high mag images in panels **a**-**d**

The Arc is primarily known for its function during feeding [2, 32, 33], but has also been implicated in mating and stress [19, 50]. Similar to the LH, the Arc displayed a significant increase in the number of c-Fos^+^ cells in response to most behaviors tested (Fig. 9; Additional file 3: Figure S3E-H.i). Significant increases in co-labeled cells (YFP^+^ and c-Fos^+^; Fig. 9a-d, A.ii-D.ii) as well as the percentage of *Dbx1*-derived cells that co-labeled with c-Fos (YFP^+^ and c-Fos^+^;/total YFP^+^; Fig. 9a-d, A.iii-D.iii) were observed in the Arc after fasting, mating and male aggression, but not after predator odor exposure. Thus, *Dbx1*-derived neurons in the Arc are activated during fasting, mating and male aggressive behaviors, but not by predator odor.

#### Ventromedial hypothalamus

**Fig. 10:**
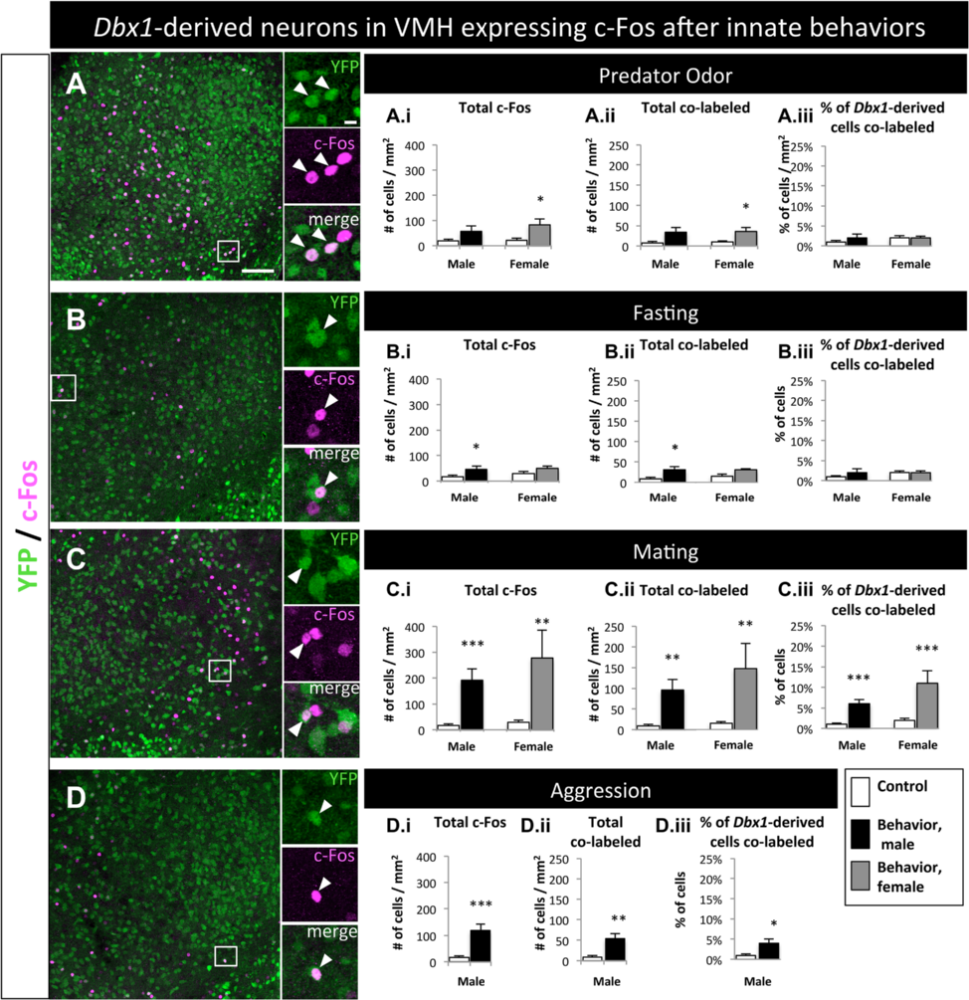
Behavioral involvement of *Dbx1*-derived cells in the VMH. **a**-**d** Images of the VMH with YFP-expressing cells co-labeled with c-Fos after mice were exposed to predator (**a**), fasting (**b**), mating (**c**), or aggression (**d**) behavioral paradigms. (A.i-D.iii) Quantification of total c-Fos (A.i-D.i), total co-labeled cells (A.ii-D.ii), and percent of *Dbx1*-derived cells co-labeled with c-Fos (YFP + c-Fos / total YFP; A.iii-D.iii) in male and female mice compared to appropriate behavioral controls. After exposure to rat odor, only females had a significant increase in total c-Fos in the Arc (A.i, *gray bar*) and only males had a significant increase in the total numbers of co-labeled cells (A.ii, *black bar*). After fasting, only males had a significant increase in total c-Fos and total co-labeled cells in the VMH (B.i and B.ii, *black bars*). Significant increases in total c-Fos, total co-labeled cells, and the percent of *Dbx1*-derived cells expressing c-Fos was detected in both males and females after mating and aggression (C.i-D.iii). Mean ± SEM; *n* = 4–13 mice per subgroup; *, *p* < 0.05; **, *p* < 0.01; ***, *p* < 0.001. The scale bar represents 50 *μ*m for low mag and 10 *μ*m high mag images in panels **a**-**d**

The VMH principally functions during mating, aggression and predator odor avoidance [1, 10, 11], but also functions in feeding [3, 33, 47]. As expected the VMH had a significant increase in c-Fos in response to mating and aggression (Fig. 10a-d, A.i-D.i; Additional file 3: Figure S3I-L.i). Significant increases in co-labeled cells (YFP^+^ and c-Fos^+^; Fig. 10a-d, A.ii-D.ii) as well as the percentage of *Dbx1*-derived cells that co-labeled with c-Fos (YFP^+^ and c-Fos^+^/total YFP^+^; Fig. 10a-d, A.iii-D.iii) were observed in the VMH after mating and male aggression. There were also significant increases in the number of c-Fos^+^ cells and *Dbx1*-derived cells expressing c-Fos after exposure to rat odor in females (Fig. 10A.i-ii) and fasting in males (Fig. 10B.ii). This suggests that the primary involvement of *Dbx1*-derived neurons in the VMH is during mating and male aggressive behaviors, but may also have minor overlapping functions with the LH and Arc during aspects of fasting and predator responses.

## Discussion

The hypothalamus is a complex multi-nucleated structure in which individual nuclei function to direct a variety of behaviors essential for survival, adaptation and species propagation [2, 3]. Despite the extensive study of hypothalamic function and anatomy, only recently has there been a greater understanding of the mechanisms of hypothalamic development, predominantly via a combination of gene expression and lineage tracing studies. Our previous study focusing on the function of the embryonic expressed homeodomain encoding transcription factor, *Dbx1* revealed *Dbx1* to have a restricted function in the specification of hypothalamic neurons required for innate stress responses but not other innate behaviors [19]. Here, we sought to extend these findings in order to provide deeper insight into the *Dbx1*-lineage contribution to diverse neuronal hypothalamic populations and to determine if *Dbx1*-derived neurons are activated by specific innate behaviors. We found a large amount of *Dbx1*-derived neuronal diversity across hypothalamic nuclei and broad activation of the *Dbx1*-lineage by different innate behaviors. Interestingly, the broad fate and generally non-selective activation of *Dbx1*-derived neurons to a variety of innate behaviors was not predicted by our previous finding of the restricted function of *Dbx1* in specification of neurons solely required for innate stress responses. However, taken together these data are consistent with a model in which there are distinct gene sets expressed during hypothalamic development that, while maybe widespread in their lineage contribution, perform select functional roles in specification of distinct hypothalamic subpopulations.

Previous fate-mapping studies of the hypothalamus have begun to provide a general understanding of the relationship between progenitor domains and mature nuclei [3, 16, 18, 28, 61]. These studies have included examination of the lineage of a variety of developmentally defined subpopulations such as those that express the transcription factors *Nkx2.1*, *Dlx*, *Nr5a1* [62, 63] and secreted factors such as *Shh* [48]. Complementing this fate-mapping work are a series of detailed and highly informative hypothalamic developmental gene expression studies [27, 28, 64]. Collectively these studies have revealed that: 1) there are gene sets that give rise to neurons and function across hypothalamic nuclei (e.gs *Rax*, *Nkx2.1*, *Asc1*) and complementary gene sets that appear to be restricted in expression and function in specification of specific nuclei (e.gs *Bsx*, *Nr5a1*) [18] and 2) consistent with our findings, embryonic gene expression domains appear to be generally predictive of the location of mature nuclei, suggesting a general lack of widespread migration across domains. This is in contrast to the telencephalon where the ventral embryonic ganglionic eminence developmental domains (MGE and CGE) give rise to immature neurons that migrate to distant areas such as the cerebral cortex and hippocampus [65–67].

Within this framework, similar to progenitors expressing the developmentally regulated genes *Shh* and *Nkx2.1*, we found that *Dbx1*^*+*^ progenitors generate a wide variety of neuronal subtypes across multiple hypothalamic nuclei. *Dbx1*-derived cells were also present, although to a lesser degree, in the VMH. In contrast, regions of the anterior hypothalamus were devoid of *Dbx1*-derived neurons. A gradient of *Dbx1*-derived cells was also observed radiating from the tuberal domain into the anterior domain, a pattern that is more pronounced in medial portions of the ventral diencephalon. This pattern of *Dbx1*-derived neuronal location was generally shared with the pattern of location of *Shh*-lineage neurons [48]. This finding perhaps reflects the overlapping embryonic expression domains of *Shh* and *Dbx1* and may further indicate putative positive control of *Dbx1* expression by Shh during forebrain development.

The hypothalamus has been thought to develop in an ‘outside-in’ manner, stemming from studies using traceable thymidine analogs indicating the lateral hypothalamic nuclei are typically born prior to medial nuclei [49, 68, 69]. More recent fate-mapping studies and analysis of molecularly defined cell types have provided another layer of complexity of development for select cell populations [48, 70]. Here, using inducible fate-mapping, we demonstrate that medial *Dbx1*-derived neurons in both LH and Arc are recombined earlier (before E9.5), with lateral populations recombined at later ages (after E9.5). Although our study is limited in that we did not conduct a birth-dating analysis of *Dbx1*-derived neurons, this observed pattern is consistent with previous *Shh* fate-mapping studies [48], and is supportive of a more complex pattern of medial-lateral development.

We further found *Dbx1*-derived neurons contribute to diverse molecularly defined populations in the LH, Arc and VMH. Within the LH, the majority of Pmch^+^ cells, which also express Nfn^+^, and Cart^+^, were *Dbx1*-derived. This finding is predicted by our loss-of-function studies in which the Pmch^+^ population was dramatically reduced [19]. In the Arc, our *Dbx1* loss-of-function studies demonstrated a ~50 % reduction of *Agrp* and *Cart* expression, with no changes in the Pomc^+^ or TH^+^ populations [19]. Here we demonstrate that ~50 % of Agrp^+^ and Cart^+^ neurons were *Dbx1*-derived. Collectively these data are consistent with a cell autonomous function of *Dbx1* in generation of LH Pmch^+^ and Arc Agrp^+^ populations. However, surprisingly, a significant proportion of Arc Pomc^+^ and TH^+^ cells were also *Dbx1*-derived. Thus, while *Dbx1*^*+*^ progenitors generate diverse populations in the Arc, it appears that *Dbx1*-independent mechanisms are required for specification of the Pomc^+^ and TH^+^ neurons.

At the behavioral level, the number of c-Fos^+^ cells in response to innate behavioral cues was increased in a predictable manner consistent with previous work [19, 55–57]. Building upon these results, we assessed the *Dbx1*-lineage contribution to these patterns of activation. We previously demonstrated that at the behavioral level conditional *Dbx1* hypothalamic loss-of-function resulted in a specific defect in innate stress responses, but not other innate behaviors such as mating or aggression [19]. Based on these findings, we anticipated that *Dbx1*-derived neurons would also be engaged (c-Fos^+^) selectively during innate stress behaviors (predator odor and fasting), but not other social behaviors (mating and aggression). In contrast, we found that across hypothalamic nuclei, *Dbx1*-derived neurons were active during multiple innate behavior tasks. Most broadly tuned to many behaviors was the LH, in which the percent of *Dbx1*-derived neurons expressing c-Fos increased after every behavioral paradigm tested. While this was not predicted by our previous loss-of-function studies, as we show here that a large portion the Pmch^+^ and Hcrt^+^ populations were *Dbx1*-derived, it is perhaps not surprising that the *Dbx1*-derived populations in the LH are responsive to a variety of innate cues.

These activation patterns, while still encompassing multiple behaviors, were more specific in the Arc and VMH. We observed an increase in the proportion of activated *Dbx1*-derived neurons after fasting, mating and male aggression in the Arc, and an increase after mating and male aggression in the VMH. In the Arc, while less than 50 % of the feeding neurons (Pomc, Agrp, and Cart) were *Dbx1*-derived, these neurons were c-Fos^+^ during fasting, likely reflecting their involvement in this major function of the Arc. In contrast, Arc *Dbx1-*derived neurons were less engaged in responses to predator odor. In the VMH, the *Dbx1*-derived neurons contributed to large portions of the ERα^+^ and Arom^+^ neuronal subpopulations, which are known to influence mating and aggressive behaviors [14, 43–45, 71]. This was reflected in the behavioral activation patterns, where the *Dbx1*-derived neurons were selectively activated during mating and aggression. While further experiments are needed to define the *Dbx1*-derived circuits that are required for specific hypothalamic-driven behaviors, these studies present novel insight into the link between developmental lineage and behavioral control.

## Conclusions

In summary, using a combination of approaches we reveal a widespread and temporally regulated contribution of *Dbx1*^*+*^ progenitors to multiple neuronal populations across hypothalamic nuclei. We further demonstrate a broad innate behavioral tuning of *Dbx1*-derived cells in the LH, Arc and VMH, implicating their involvement in multiple innate behaviors. Thus, our studies provide new information regarding the link between hypothalamic embryonic gene expression patterns, postnatal neuronal fate, subtype identity and potential contribution to essential hypothalamic-driven behaviors.

## Additional files

Additional file 1: Figure S1. Correspondence between *Dbx1*-driven recombination and *Dbx1* expression. Regions of recombination in the tuberal hypothalamus (schematic on left) at the level of the E11.5 lateral hypothalamic primordium and (A-C) and E12.5 arcuate nucleus primordium (E-G) are shown. Filled arrowheads highlight recombined *Dbx1*-derived YFP+ cells (A, C, E, G). Open arrowheads highlight Dbx1+ progenitors (B, F, C, G). A small number of YFP+/Dbx1+ double-labeled cells are observed (boxed area in G and high magnification shown on right panels). The low number of double-labeled cells is likely due to the time lag between the transient expression of *Dbx1* in dividing progenitors and YFP expression resulting from Cre-driven recombination. Abbreviation: 3^rd^ V (3^rd^ ventricle). (TIF 44748 kb) Additional file 2: Figure S2.
*Dbx1*-derived cells in nuclei as defined by specific markers. (A-D) Schematic of medial (top) to lateral (bottom) sagittal views of the embryonic forebrain. (A.i-D.i) As shown by YFP expression in E13.5 *Dbx1* ^*Cre*^ *;Rosa26YFP* embryos, *Dbx1*-derived cells are are found in primordial hypothalamic nuclei including in the paraventricular, arcuate, ventral medial and lateral progenitor domains. (A.ii-D.iii) As shown by ISH of serial sections, specific markers of the paraventriclar (*Sim1,* A.ii; *Fezf1,* A.iii), arcuate (*Pomc*, B.ii; *Bsx,* B.iii), ventral medial (*Fezf1,* C.ii; *Nr5a1,* C.iii) and lateral hypothalamic (*Pmch,* D.ii; *Lhx9,* D.iii) nuclei overlap with expression of YFP. The scale bar represents 500 *μ*m. (TIF 157696 kb) Additional file 3: Figure S3. Increased c-Fos in Arc, LH, and VMH after innate behaviors. (A-D.i) Images of the c-Fos expression in the Arc (A-D), LH (E-H), and VMH (I-L) after mice were exposed to mating (A, E, and I), aggression (B, F, and J), fasting (C, G, and K), or predator (D, H, and L) behavioral paradigms. The number of cells expressing c-Fos in the Arc, LH, and VMH are increased after exposure to the four behavioral paradigms. The scale bar represents 500 *μ*m in the LH and VMH (A-D.i, I-L.i) and 250 *μ*m in the Arc (E-H.i). (TIF 172032 kb)

## References

[1] Gross CT, Canteras NS (2012). The many paths to fear. Nat Rev Neurosci.

[2] Sternson SM (2013). Hypothalamic survival circuits: blueprints for purposive behaviors. Neuron.

[3] Elson AE, Simerly RB. Developmental specification of metabolic circuitry. Front Neuroendocrinol. 2015. doi:10.1016/j.yfrne.2015.09.003. 10.1016/j.yfrne.2015.09.003 PMC4681622 26407637

[4] Caqueret A, Yang C, Duplan S, Boucher F, Michaud JL (2005). Looking for trouble: a search for developmental defects of the hypothalamus. Horm Res.

[5] Grossman SP (1975). Role of the hypothalamus in the regulation of food and water intake. Psychol Rev.

[6] O’Rahilly S (2009). Human genetics illuminates the paths to metabolic disease. Nature.

[7] Morton GJ, Cummings DE, Baskin DG, Barsh GS, Schwartz MW (2006). Central nervous system control of food intake and body weight. Nature.

[8] Saper CB (2006). Staying awake for dinner: hypothalamic integration of sleep, feeding, and circadian rhythms. Prog Brain Res.

[9] Brown JA, Woodworth HL, Leinninger GM (2015). To ingest or rest? Specialized roles of lateral hypothalamic area neurons in coordinating energy balance. Front Syst Neurosci.

[10] Saper CB, Lowell BB (2014). The hypothalamus. Curr Biol.

[11] Falkner AL, Lin D (2014). Recent advances in understanding the role of the hypothalamic circuit during aggression. Front Syst Neurosci.

[12] Burdakov D, Alexopoulos H (2004). Metabolic state signalling through central hypocretin/orexin neurons. Eur J Neurosci.

[13] Xu X, Coats JK, Yang CF, Wang A, Ahmed OM, Alvarado M, Izumi T, Shah NM (2012). Modular genetic control of sexually dimorphic behaviors. Cell.

[14] Lee H, Kim DW, Remedios R, Anthony TE, Chang A, Madisen L, Zeng H, Anderson DJ (2014). Scalable control of mounting and attack by Esr1+ neurons in the ventromedial hypothalamus. Nature.

[15] Nomoto K, Lima SQ (2015). Enhanced male-evoked responses in the ventromedial hypothalamus of sexually receptive female mice. Curr Biol.

[16] Chatterjee M, Li JY (2012). Patterning and compartment formation in the diencephalon. Front Neurosci.

[17] Sokolowski K, Corbin JG (2012). Wired for behaviors: from development to function of innate limbic system circuitry. Front Mol Neurosci.

[18] Bedont JL, Newman EA, Blackshaw S (2015). Patterning, specification, and differentiation in the developing hypothalamus. Wiley Interdiscip Rev Dev Biol.

[19] Sokolowski K, Esumi S, Hirata T, Kamal Y, Tran T, Lam A, Oboti L, Brighthaupt SC, Zaghlula M, Martinez J, Ghimbovschi S, Knoblach S, Pierani A, Tamamaki N, Shah NM, Jones KS, Corbin JG (2015). Specification of select hypothalamic circuits and innate behaviors by the embryonic patterning gene Dbx1. Neuron.

[20] Pierani A, Moran-Rivard L, Sunshine MJ, Littman DR, Goulding M, Jessell TM (2001). Control of interneuron fate in the developing spinal cord by the progenitor homeodomain protein Dbx1. Neuron.

[21] Hirata T, Li P, Lanuza GM, Cocas LA, Huntsman MM, Corbin JG (2009). Identification of distinct telencephalic progenitor pools for neuronal diversity in the amygdala. Nat Neurosci.

[22] Vue TY, Aaker J, Taniguchi A, Kazemzadeh C, Skidmore JM, Martin DM, Martin JF, Treier M, Nakagawa Y (2007). Characterization of progenitor domains in the developing mouse thalamus. J Comp Neurol.

[23] Lu S, Bogarad LD, Murtha MT, Ruddle FH (1992). Expression pattern of a murine homeobox gene, Dbx, displays extreme spatial restriction in embryonic forebrain and spinal cord. Proc Natl Acad Sci U S A.

[24] Shoji H, Ito T, Wakamatsu Y, Hayasaka N, Ohsaki K, Oyanagi M, Kominami R, Kondoh H, Takahashi N (1996). Regionalized expression of the Dbx family homeobox genes in the embryonic CNS of the mouse. Mech Dev.

[25] Flames N, Pla R, Gelman DM, Rubenstein JL, Puelles L, Marín O (2007). Delineation of multiple subpallial progenitor domains by the combinatorial expression of transcriptional codes. J Neurosci.

[26] Causeret F, Ensini M, Teissier A, Kessaris N, Richardson WD, Lucas de Couville T, Pierani A (2011). Dbx1-expressing cells are necessary for the survival of the mammalian anterior neural and craniofacial structures. PLoS One.

[27] Kurrasch DM, Cheung CC, Lee FY, Tran PV, Hata K, Ingraham HA (2007). The neonatal ventromedial hypothalamus transcriptome reveals novel markers with spatially distinct patterning. J Neurosci.

[28] Shimogori T, Lee DA, Miranda-Angulo A, Yang Y, Wang H, Jiang L, Yoshida AC, Kataoka A, Mashiko H, Avetisyan M, Qi L, Qian J, Blackshaw S (2010). A genomic atlas of mouse hypothalamic development. Nat Neurosci.

[29] Verret L, Goutagny R, Fort P, Cagnon L, Salvert D, Léger L, Boissard R, Salin P, Peyron C, Luppi PH. A role of melanin-concentrating hormone producing neurons in the central regulation of paradoxical sleep. BMC Neurosci. 2003;4:19. 10.1186/1471-2202-4-19 PMC201018 12964948

[30] Burdakov D, Luckman SM, Verkhratsky A (2005). Glucose-sensing neurons of the hypothalamus. Philos Trans R Soc Lond B Biol Sci.

[31] Maniam J, Morris MJ (2012). The link between stress and feeding behaviour. Neuropharmacology.

[32] Yeo GS, Heisler LK (2012). Unraveling the brain regulation of appetite: lessons from genetics. Nat Neurosci.

[33] Sohn JW, Elmquist JK, Williams KW (2013). Neuronal circuits that regulate feeding behavior and metabolism. Trends Neurosci.

[34] Elias CF, Lee CE, Kelly JF, Ahima RS, Kuhar M, Saper CB, Elmquist JK (2001). Characterization of CART neurons in the rat and human hypothalamus. J Comp Neurol.

[35] Fort P, Salvert D, Hanriot L, Jego S, Shimizu H, Hashimoto K, Mori M, Luppi PH (2008). The satiety molecule nesfatin-1 is co-expressed with melanin concentrating hormone in tuberal hypothalamic neurons of the rat. Neuroscience.

[36] Croizier S, Franchi-Bernard G, Colard C, Poncet F, La Roche A, Risold PY (2010). A comparative analysis shows morphofunctional differences between the rat and mouse melanin-concentrating hormone systems. PLoS One.

[37] Sundvik M, Kudo H, Toivonen P, Rozov S, Chen YC, Panula P (2011). The histaminergic system regulates wakefulness and orexin/hypocretin neuron development via histamine receptor H1 in zebrafish. FASEB J.

[38] Dalal J, Roh JH, Maloney SE, Akuffo A, Shah S, Yuan H, Wamsley B, Jones WB, Strong C, Gray PA (2013). Translational profiling of hypocretin neurons identifies candidate molecules for sleep regulation. Genes Dev.

[39] Chan-Palay V, Záborszky L, Köhler C, Goldstein M, Palay SL (1984). Distribution of tyrosine-hydroxylase-immunoreactive neurons in the hypothalamus of rats. J Comp Neurol.

[40] Broberger C (1999). Hypothalamic cocaine- and amphetamine-regulated transcript (CART) neurons: histochemical relationship to thyrotropin-releasing hormone, melanin-concentrating hormone, orexin/hypocretin and neuropeptide Y. Brain Res.

[41] Ovesjö ML, Gamstedt M, Collin M, Meister B (2001). GABAergic nature of hypothalamic leptin target neurones in the ventromedial arcuate nucleus. J Neuroendocrinol.

[42] Lee B, Kim SG, Kim J, Choi KY, Lee S, Lee SK, Lee JW (2013). Brain-specific homeobox factor as a target selector for glucocorticoid receptor in energy balance. Mol Cell Biol.

[43] Sano K, Tsuda MC, Musatov S, Sakamoto T, Ogawa S (2013). Differential effects of site-specific knockdown of estrogen receptor α in the medial amygdala, medial pre-optic area, and ventromedial nucleus of the hypothalamus on sexual and aggressive behavior of male mice. Eur J Neurosci.

[44] Yang CF, Chiang MC, Gray DC, Prabhakaran M, Alvarado M, Juntti SA, Unger EK, Wells JA, Shah NM. Sexually dimorphic neurons in the ventromedial hypothalamus govern mating in both sexes and aggression in males. Cell. 2013;153:896–909. doi:10.1016/j.cell.2013.04.017. 10.1016/j.cell.2013.04.017 PMC3767768 23663785

[45] Dugger BN, Morris JA, Jordan CL, Breedlove SM (2007). Androgen receptors are required for full masculinization of the ventromedial hypothalamus (VMH) in rats. Horm Behav.

[46] Danielian PS, Muccino D, Rowitch DH, Michael SK, McMahon AP (1998). Modification of gene activity in mouse embryos in utero by a tamoxifen-inducible form of Cre recombinase. Curr Biol.

[47] Zervas M, Millet S, Ahn S, Joyner AL (2004). Cell behaviors and genetic lineages of the mesencephalon and rhombomere 1. Neuron.

[48] Alvarez-Bolado G, Paul FA, Blaess S (2012). Sonic hedgehog lineage in the mouse hypothalamus: from progenitor domains to hypothalamic regions. Neural Dev.

[49] Risold PY, Croizier S, Legagneux K, Brischoux F, Fellmann D, Griffond B (2009). The development of the MCH system. Peptides.

[50] Yang S, Lee Y, Voogt JL (1999). Fos expression in the female rat brain during the proestrous prolactin surge and following mating. Neuroendocrinology.

[51] Atasoy D, Betley JN, Su HH, Sternson SM (2012). Deconstruction of a neural circuit for hunger. Nature.

[52] Betley JN, Cao ZF, Ritola KD, Sternson SM (2013). Parallel, redundant circuit organization for homeostatic control of feeding behavior. Cell.

[53] Cohen RS, Pfaff DW (1992). Ventromedial hypothalamic neurons in the mediation of long-lasting effects of estrogen on lordosis behavior. Prog Neurobiol.

[54] Silva BA, Mattucci C, Krzywkowski P, Murana E, Illarionova A, Grinevich V, Canteras NS, Ragozzino D, Gross CT (2013). Independent hypothalamic circuits for social and predator fear. Nat Neurosci.

[55] Lin D, Boyle MP, Dollar P, Lee H, Lein ES, Perona P, Anderson DJ (2011). Functional identification of an aggression locus in the mouse hypothalamus. Nature.

[56] Canteras NS, Chiavegatto S, Ribeiro do Valle LE, Swanson LW (1997). Severe reduction of rat defensive behavior to a predator by discrete hypothalamic chemical lesions. Brain Res Bull.

[57] Beijamini V, Guimarães FS (2006). c-Fos expression increase in NADPH-diaphorase positive neurons after exposure to a live cat. Behav Brain Res.

[58] García AP, Aitta-aho T, Schaaf L, Heeley N, Heuschmid L, Bai Y, Apergis-Schoute J. Nicotinic α4 receptor-mediated cholinergic influences on food intake and activity patterns in hypothalamic circuits. PLoS One. 2015;10(8):e0133327. doi:10.1371/journal.pone.0133327. 10.1371/journal.pone.0133327 PMC4527587 26247203

[59] Dragunow M, Faull R (1989). The use of c-fos as a metabolic marker in neuronal pathway tracing. J Neurosci Methods.

[60] Bullitt E (1990). Expression of c-fos-like protein as a marker for neuronal activity following noxious stimulation in the rat. J Comp Neurol.

[61] Hoch RV, Rubenstein JL, Pleasure S (2009). Genes and signaling events that establish regional patterning of the mammalian forebrain. Semin Cell Dev Biol.

[62] Yee CL, Wang Y, Anderson S, Ekker M, Rubenstein JL (2009). Arcuate nucleus expression of NKX2.1 and DLX and lineages expressing these transcription factors in neuropeptide Y(+), proopiomelanocortin(+), and tyrosine hydroxylase(+) neurons in neonatal and adult mice. J Comp Neurol.

[63] Dhillon H, Zigman JM, Ye C, Lee CE, McGovern RA, Tang V, Kenny CD, Christiansen LM, White RD, Edelstein EA, Coppari R, Balthasar N, Cowley MA, Chua S, Elmquist JK, Lowell BB (2006). Leptin directly activates SF1 neurons in the VMH, and this action by leptin is required for normal body-weight homeostasis. Neuron.

[64] Ferran JL, Puelles L, Rubenstein JL (2015). Molecular codes defining rostrocaudal domains in the embryonic mouse hypothalamus. Front Neuroanat.

[65] Batista-Brito R, Fishell G (2009). The developmental integration of cortical interneurons into a functional network. Curr Top Dev Biol.

[66] Nery S, Fishell G, Corbin JG (2002). The caudal ganglionic eminence is a source of distinct cortical and subcortical cell populations. Nat Neurosci.

[67] Anderson SA, Marín O, Horn C, Jennings K, Rubenstein JL (2001). Distinct cortical migrations from the medial and lateral ganglionic eminences. Development.

[68] Shimada M, Nakamura T (1973). Time of neuron origin in mouse hypothalamic nuclei. Exp Neurol.

[69] Markakis EA, Swanson LW (1997). Spatiotemporal patterns of secretomotor neuron generation in the parvicellular neuroendocrine system. Brain Res Rev.

[70] Padilla SL, Carmody JS, Zeltser LM (2010). Pomc-expressing progenitors give rise to antagonistic neuronal populations in hypothalamic feeding circuits. Nat Med.

[71] Imwalle DB, Scordalakes EM, Rissman EF (2002). Estrogen receptor alpha influences socially motivated behaviors. Horm Behav.

