# Supplementary figures and images for: Molecular and behavioral profiling of Dbx1-derived neurons in the arcuate, lateral and ventromedial hypothalamic nuclei
Source: Neural Dev. 2016 May 21;11:12. doi: 10.1186/s13064-016-0067-9 (PMC4875659; doi:10.1186/s13064-016-0067-9)

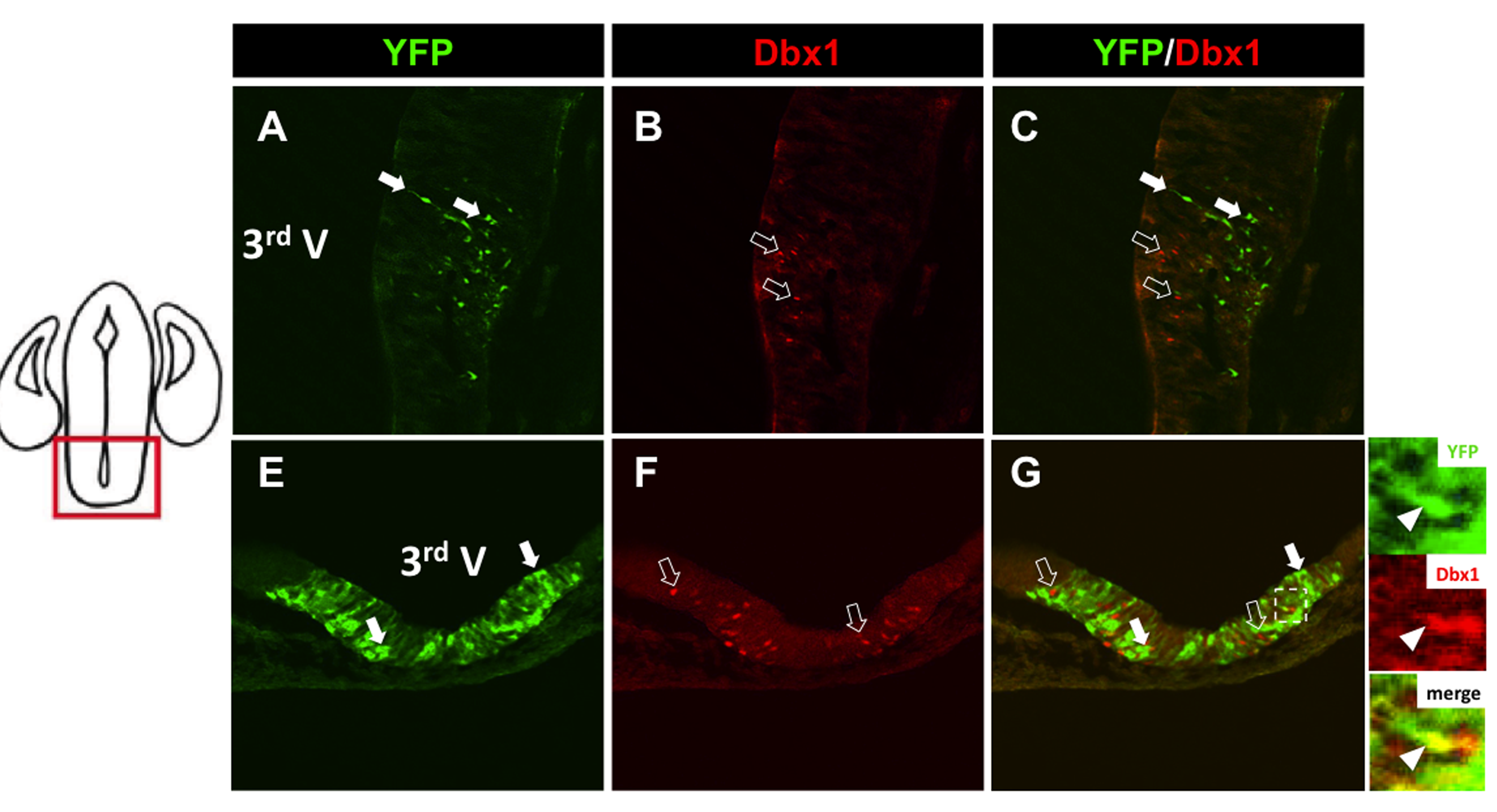

Supplement: Additional file 1: Figure S1. — Correspondence between Dbx1-driven recombination and Dbx1 expression. Regions of recombination in the tuberal hypothalamus (schematic on left) at the level of the E11.5 lateral hypothalamic primordium and (A-C) and E12.5 arcuate nucleus primordium (E-G) are shown. Filled arrowheads highlight recombined Dbx1-derived YFP+ cells (A, C, E, G). Open arrowheads highlight Dbx1+ progenitors (B, F, C, G). A small number of YFP+/Dbx1+ double-labeled cells are observed (boxed area in G and high magnification shown on right panels). The low number of double-labeled cells is likely due to the time lag between the transient expression of Dbx1 in dividing progenitors and YFP expression resulting from Cre-driven recombination. Abbreviation: 3rd V (3rd ventricle). (TIF 44748 kb) [file 13064_2016_67_MOESM1_ESM.tif]
